# Authenticating Health Activity Data Using Distributed Ledger Technologies

**DOI:** 10.1016/j.csbj.2018.06.004

**Published:** 2018-07-17

**Authors:** James Brogan, Immanuel Baskaran, Navin Ramachandran

**Affiliations:** a University College London, Centre for Health Informatics & Multiprofessional Education, London, United Kingdom; b University College London Computer Science, London, United Kingdom; c Albert Einstein College of Medicine, Bronx, United States

**Keywords:** Activity data, Blockchain, Distributed ledger technologies, eHealth, Medical sensors, Remote monitoring, Wearable devices

## Abstract

The on-demand digital healthcare ecosystem is on the near horizon. It has the potential to extract a wealth of information from “big data” collected at the population level, to enhance preventive and precision medicine at the patient level. This may improve efficiency and quality while decreasing cost of healthcare delivered by professionals. However, there are still security and privacy issues that need to be addressed before algorithms, data, and models can be mobilized safely at scale. In this paper we discuss how distributed ledger technologies can play a key role in advancing electronic health, by ensuring authenticity and integrity of data generated by wearable and embedded devices. We demonstrate how the Masked Authenticated Messaging extension module of the IOTA protocol can be used to securely share, store, and retrieve encrypted activity data using a tamper-proof distributed ledger.

## 1 Introduction

### 1.1 Electronic and Mobile Health

We are moving towards a future where information and communication technologies for health will be ubiquitous in the daily lives of most patients. The fusion of electronic processes with mobile and embedded devices has the potential to improve access, efficiency, and quality of personalized care [1, 2]. Wearable and embedded devices (such as pacemakers, smart glucometers, and activity trackers) paired with remote monitoring and telemedicine services, will allow patients to receive care and maintain their health with minimal disruption of their day-to-day activities. While the implementation of these cyber-physical systems can greatly benefit society as a whole, the security and privacy of each patient must be ensured.

To achieve the vision of an increasingly distributed healthcare system, algorithmic processing of large volumes of data will train models that continuously monitor health metrics, and report or act on abnormalities in real time. The continued development of efficacious models and algorithms is critical for the improvement of healthcare, and should be an open and vetted process within the medical community. As these shared models and algorithms are improved over time, devices may submit and receive over-the-air updates in a federated manner [3].

It is imperative that this process is secure, and the data used to train models should be immutable, and verifiable. This would help gain the trust of clinicians and patients, and therefore aid in mobilizing data for analysis. In order to realize this verifiable future, healthcare technologists will have to reach beyond current tools. The emergence of distributed ledger technologies offers promise to bridge this gap.

### 1.2 Distributed Ledger Technologies

A distributed ledger is a distributed database, maintained by a consensus protocol run by nodes in a peer-to-peer network. This consensus protocol replaces a central administrator, since all peers contribute to maintaining the integrity of the database. This absence of a central controller means that individuals may regain agency over their data.

**Fig. 1:**
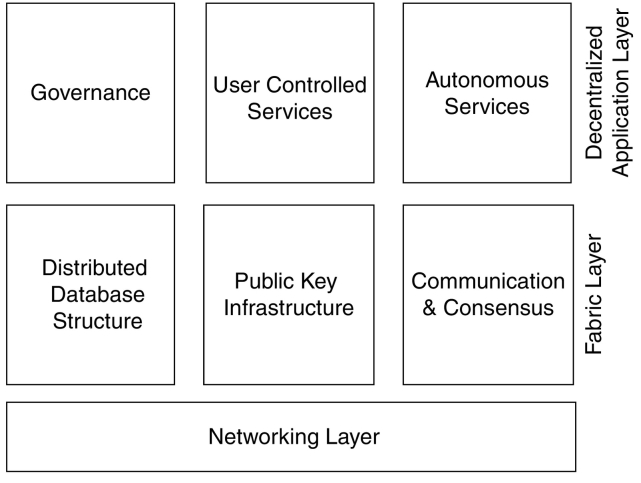
Layers of a distributed ledger system.

Distributed ledger technologies can be broken down into two fundamental layers, as displayed in Fig. 1 [4]. The first layer is the *fabric* layer, which is the code base for communication, consensus, public key infrastructure, and database structuring. The second layer is the *application* layer. This layer can contain logic, and is open for anyone to create novel decentralized applications that run on top of the *fabric* layer. It is important to understand that the individuals who develop and maintain the fabric layer are in control of the system's core functionality. Therefore, there is a level of centralization associated with this technology. However, there are concepts for decentralized governance models that sit in the application layer, and enable network participants to have a say in future updates to the fabric layer.

While distributed ledgers allow any peer to create new transactions and read from the shared database, malicious changes to historical transactions are spotted by honest peers. This makes it difficult for an adversary to alter a transaction after it has been accepted by all network participants. In this way, all peers can freely read and write to the distributed ledger without the concern of an adversary corrupting historical data stored on the distributed ledger.

**Fig. 2:**
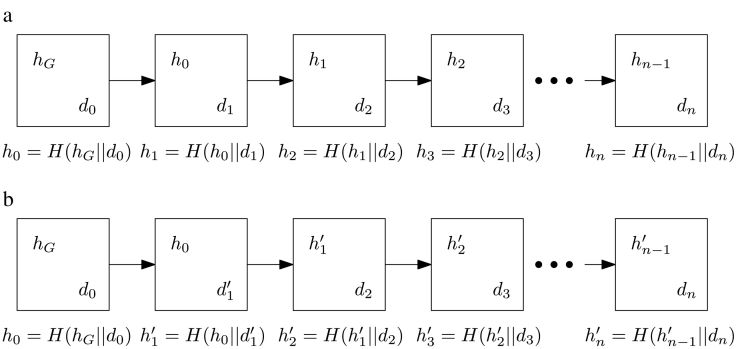
Relations between blocks of data are established in a) by linking them together using a one-way hash function, *H*. If data, *d*_1_, is altered in the second block of b), the hashes of all subsequent blocks are altered.

This trustless environment is made possible because of modern cryptographic primitives, such as one-way hash functions, and distributed consensus protocols that comprise part of the *fabric* [5]. All transactions that are written to a distributed ledger are cryptographically connected to existing transactions in a unique way that is easily verifiable. These relations establish chronology and trust between peers since any alterations in archived transactions are propagated through the ledger, as shown in Fig. 2.

Specialized distributed consensus protocols enable databases to be shared in a peer-to-peer network without the need for all participants to trust each other. Distributing databases between multiple peers in a trustless fashion has enabled novel decentralized applications such as cryptographic currencies and smart contracts [[6], [7], [8]]. Despite the promise of this emerging technology, current protocols face a trade-off between latency and transaction finality. Common practice is to wait for six blocks to be added to the longest chain before reaching a high level of confidence that a transaction is final on the Bitcoin network. This is equivalent to about an hour long wait [9]. Applications that require exchange of value and low latency cannot be certain that their transactions are final in a shorter time frame, and must trust the payer to not double spend [10].

The current incentive schemes that allow these protocols to spread virally make inefficient use of computational resources while constraining the transaction rate on the network. The Bitcoin protocol currently supports less than 4 transactions per second in the whole network, with each transaction requiring a fee that may exceed $1 [11, 12]. Similarly, the Ethereum protocol currently processes less than 10 transactions per second across the entire network, with each transaction accruing a fee of at least $0.50 [13, 14]. To use a distributed ledger at scale for financial or other industrial use cases, this low throughput and high fee model will not suffice.

There has been an increasing amount of research in the field of distributed systems stemming from the Byzantine Generals Problem described by Lamport, Shostak and Pease [15]. Various network topologies and distributed consensus protocols that maintain the integrity of a distributed ledger while facilitating high transaction throughputs as well as zero transaction fees have been explored. A few notable protocols that claim to achieve the aforementioned properties are *Algorand*, *IOTA*, *hashgraph*, and *Ouroboros* [[16], [17], [18], [19]]. While we see this technology is very promising for the future of electronic finance, it actually has a place in every industry that is data driven. In the remainder of this paper we focus on a healthcare-centric application of IOTA, a feeless, permissionless distributed ledger protocol that aims to overcome the scalability issues faced by earlier iterations of distributed ledger technologies.

### 1.3 Objectives

This study builds on previous works that describe frameworks for how blockchain technology and Fast Healthcare Interoperability Resources (FHIR) could foster the growth of a decentralized patient data store, such as a Health Information Exchange network. While these works describe the value of blockchain in healthcare, and focus on network architecture and consensus in the *fabric* layer, we take a more granular approach [[20], [21], [22], [23]]. This paper focuses on the transport of health activity data generated by wearable and embedded devices to a distributed ledger. In particular, we employ Masked Authenticated Messaging (MAM) from the *application* layer of the IOTA stack to encrypt, authenticate, and broadcast activity data structured as FHIR observation resources to the IOTA network [24]. This new model has the potential to restore patient agency over their health activity data, and improve data sharing capabilities across many facets of the digital healthcare ecosystem.

In Section 2, we introduce the IOTA protocol and its MAM extension module. We then describe our implementation in Section 3. In Section 4, we provide an overview of authenticated data structures, and their theoretical underpinnings. In the remainder of the paper, we present and discuss the outcome of our case study, as well as plans for future work.

## 2 Background

### 2.1 IOTA

**Fig. 3:**
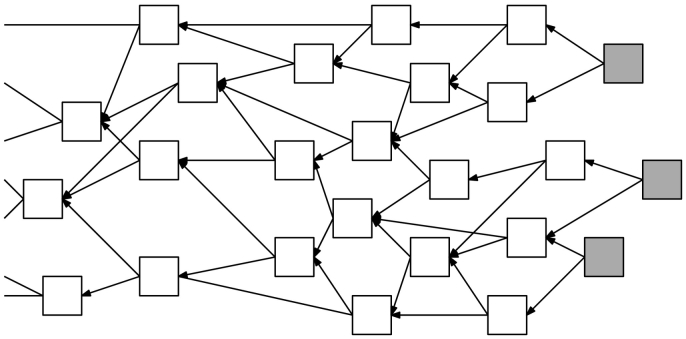
The tangle is a directed acyclic graph (DAG) that requires every incoming transaction to verify two unapproved transactions in order to successfully attach to the graph. White squares represent approved transactions, while gray squares represent unapproved transactions.

The IOTA protocol was designed to be lightweight and malleable, serving as the backbone for secure data communication between Internet-of-Things (IoT) devices. It differentiates itself from traditional blockchain-based distributed ledger protocols by addressing two major pain points: latency and fees. IOTA does not utilize the concept of blocks and miners. Instead, all transactions that want to be added to IOTA's distributed ledger, known as the tangle, must validate two unconfirmed transactions on the ledger by solving a low-cost computational puzzle similar to hashcash [25]. This mechanism enables a novel architecture with a scalable approach to transaction confirmation rates as displayed in Fig. 3. In addition, the IOTA protocolâ€™s security and operation model was designed with bandwidth constrained environments and quantum computers in mind. Therefore, hash-based signatures align well with the protocol's design goals. The Winternitz one-time signature scheme implemented within the IOTA protocol provides resistance to quantum computers, and allows for efficient broadcast authentication in sensor networks due to the low power requirements for computation and communication [26, 27].

Since there are no fees to broadcast a transaction to the network and persist it to the tangle, it is feasible to use the IOTA protocol to ensure data integrity over time. IOTA offers an extension called MAM, an experimental module acting as a second layer data communication protocol [[28], [29], [30]]. MAM extends the functionality of IOTA transactions to enable new applications on top of the *fabric* layer by encrypting and authenticating data streams. Therefore, users can broadcast and retrieve encrypted, authenticated data streams being transmitted through the tangle as zero-value transactions.

Two key features of the current implementation of the MAM extension are post-quantum cryptography, and forward transaction linking. Post-quantum cryptographic algorithms are thought to be secure against an attack by a sufficiently strong quantum computer. This is not true of many cryptographic algorithms that are currently used to encrypt messages that travel across the Internet today, and will be of paramount importance when transporting sensitive data in the future.

**Fig. 4:**
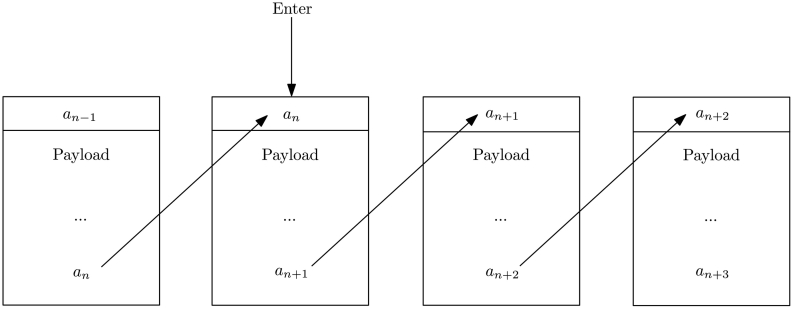
Forward transaction linking. Given the address *a*_*n*_, a subscriber can locate the transaction and retrieve the address of the next transaction, *a*_*n*+1_. This enables subscribers to retrieve transactions at or after their point of entry, but not before their point of entry.

Forward transaction linking in MAM is analogous to the data structure known as singly-linked lists. Given transaction *n*, one has the pointer to transaction *n* + 1. However, they have no knowledge of the location of transaction *n* − 1. Therefore, they cannot read any messages in a stream of data *before* their point of entry. This enables forward secrecy, which is the notion of only being able to read future transactions (Fig. 4). These features allow MAM to be applied to a wide variety of applications ranging from over-the-air sensor updates, to data marketplaces and identity management.

### 2.2 The Relationship Between IOTA Transactions and Masked Authenticated Messages

The IOTA tangle is maintained and augmented by a network of nodes running the IOTA Reference Implementation (IRI) and communicating over a JSON-REST HTTP^1^ interface [31]. The functional unit of the IOTA tangle is the transaction. The current anatomy of a transaction is given in [32].

Each field of a transaction object serves a single purpose, with the exception of the *signatureMessageFragment*. This field can hold up to 2187 trytes,^2^ and contains either a user's digital signature for a value-based transaction, or user-defined data for a zero-value transaction issued on the IOTA network. The ability to store user-defined data in this field opens the door for the tangle to serve as a tamper-proof, permissionless data repository.

Data transmitted using MAM is encrypted, or masked, and assigned to the *signatureMessageFragment* field of transaction objects. These transaction objects are then stored and retrieved via the IOTA tangle. As a result, de-identified health data can be encrypted and stored on a tamper-proof ledger that is distributed across many peers. This fills an important need in healthcare, where access, integrity and privacy meet.

Data payloads can be broadcasted using the MAM module at any time, and only require a small amount of proof of work to be propagated through the network. In theory, these messages can have any size. It is important to understand that this does not mean that every bit of data should be transmitted using MAM. For instance, it may be useful to broadcast wearable device data points using MAM to ensure integrity of an encrypted data stream generated and stored outside of the supervision of healthcare professionals. However, it may not make sense to broadcast large medical imaging files that are generated, stored, transmitted, and accessed by healthcare professionals. Other means of ensuring integrity of large files may be more appropriate.

**Fig. 5:**
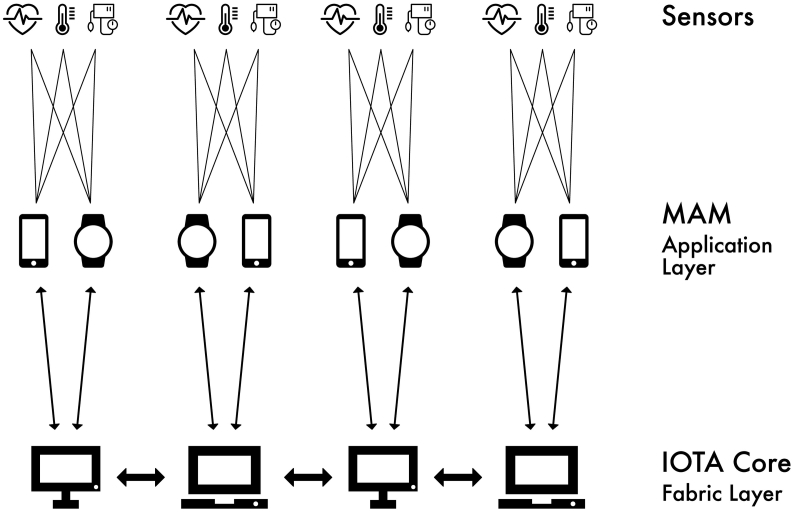
High-level system overview. Sensors are embedded in an IoT device, or interact with an IoT device via bluetooth low energy (BLE) or Long Range (LoRA) protocols. MAM clients running on IoT devices in the application layer process data from sensors. Such devices are capable of performing proof-of-work, but cannot process the whole ledger. This application layer may alternatively run on the full nodes. Full nodes comprise the fabric layer, and are capable of processing and storing the current state of the ledger.

MAM messages can be *attached* to and *fetched* from the tangle, using a client that communicates with a full node running the *fabric* layer (Fig. 5). This means that IoT devices capable of running a MAM client will be able to emit encrypted data streams using this communication protocol. For instance, a wearable device that is not fit to store the current state of the IOTA tangle can still broadcast data using MAM.

### 2.3 MAM Channels and Modes

The MAM extension module can be thought of as an internet radio with variable levels of security. The entity that wants to broadcast messages must have a seed. A seed is a master identifier that is kept secret by its owner. From the seed, the owner can deterministically produce addresses and signatures. Likewise, the owner can generate channels used in the MAM extension module. Only an entity that possesses the seed will be able to deterministically generate these addresses, signatures, and channels.

Once a channel is created, the owner can encrypt messages with the channel key and broadcast them on the tangle. Only users that know the MAM channel key can decode the message after *fetching* this transaction from the tangle. Messages broadcasted on the same channel are linked in chronological order. This allows the notion of forward secrecy, where parties that gain access to a channel cannot look back through the history of the channel before their entrance.

MAM enables three modes of privacy that control visibility and access to channels: public, private, and restricted (Table 1). In each mode, the MAM channel's ID is the address of the transaction on the tangle. This allows a simple query of the tangle to return a MAM transaction. However, the key used to decode the payload contained within the MAM transaction need not be equal to the MAM channel ID. Another useful property of MAM is transaction linking. When a user decodes a payload, they receive the message and the channel key for the next payload. As we will see below, this feature comes in handy for public and private modes.

**Table 1:** MAM channel modes**.** Summary table displaying the relationship between the MAM channel ID, channel key (CK), and authorization key (AK). In restricted mode, both the authorization key and channel key are required to decrypt the MAM data payload.

Mode	Channel ID	Decryption key	Authorization key
Public	CK	CK	–
Private	*H*(CK)	CK	–
Restricted	*H*(AK‖CK)	CK + AK	AK

In the case of public mode, the transaction address is both the channel ID and channel key. Therefore, anyone on the network can read all of the contents of the message chain. In private mode, there is an added layer of security that prevents unauthorized entities from reading a message chain. The transaction address is the channel ID, which is the *hash* of the channel key. Therefore, the broadcaster has to securely communicate the channel key to all entities subscribing so they can locate the message on the tangle. These subscribers query the tangle for the hash of the channel key, and decode the message payload using the channel key. If an adversary were to come across a MAM transaction that was sent using private mode, they could not decrypt the message payload using the channel ID because it is the hash of the channel key, not the key itself. Since preimage attacks are computationally infeasible, it would be very difficult for an adversary to reproduce the channel key from the channel ID. However, producing the channel ID from the channel key is trivial for subscribers.

**Fig. 6:**
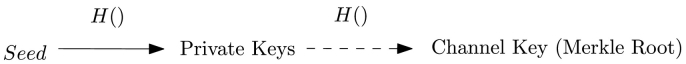
MAM channel key generation. A one-way hash function, *H*, is used to generate private keys from a user's seed for their one-time signature scheme. The private keys are then hashed according to the Merkle Hash Technique in Section 4.2 to produce the Merkle Root. The root of the tree serves as the channel key.

In restricted mode, the transaction address is the *hash* of the authorization key concatenated with the channel key. Both the channel key and authorization key are also required to decode the message payload. The authorization key is specified by the broadcaster, and can be changed at any point in the channels stream. This gives the broadcaster the power to revoke access from future messages in their channel at any point in time by changing the authorization key. In this case, a subscriber who no longer has access to the current authorization key would not be able to locate or decode future messages in the stream. Therefore, this method could be used to terminate access for subscribers after a certain point in a stream. A simplified depiction of the relationship between elements used to generate a MAM channel is presented in Fig. 6. In the following section we present the design of our implementation.

## 3 Methodology

### 3.1 Broadcasting and Retrieving Real-Time Activity Data From Wearables Through the Tangle

Given MAM as a lightweight data transmission protocol over a tamper-proof distributed ledger, we set out to assess its potential for broadcasting sensitive activity data. We implemented a system that could broadcast sensor data from wearable devices using MAM. We used the MAM JavaScript wrapper and populated MAM data payloads with FHIR observation resources structured in JSON format [33, 34]. The FHIR observations were coded using Logical Observation Identifiers Names and Codes (LOINC) to enable structural and semantic interoperability [25, 35].

Each device was configured to transmit data through a restricted MAM channel with access controls that could be defined by a patient at the transaction level. If a patient would like to grant access to one or more physicians, they could send their channel keys to the physician(s). In return, the physician could retrieve and authenticate the associated data stream(s) from the tangle. If a patient would like to revoke access to their data stream at any time, they could simply update their MAM channel's authorization key and provide it to a desired subset of their health care providers.

**Fig. 7:**
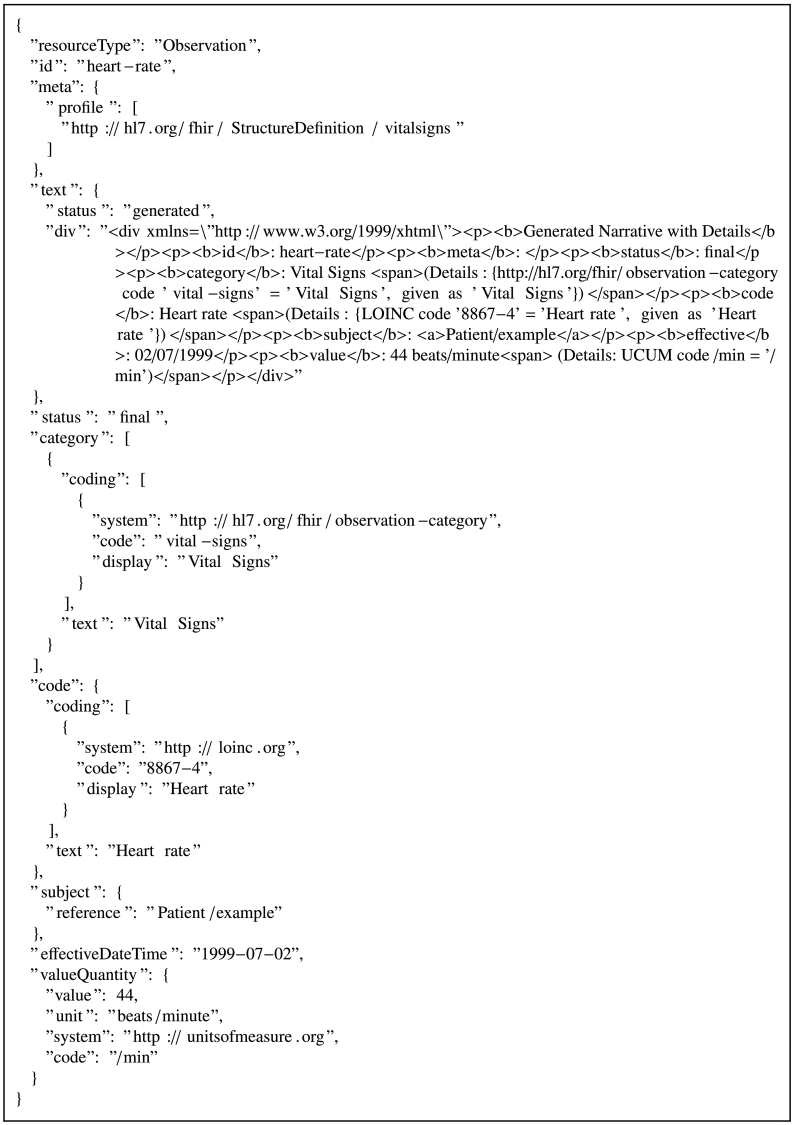
FHIR observation resource for heart rate in JSON [36].

With this system in place, a wearable device^3^ automatically broadcasted real-time data to the tangle through a smart phone application using the MAM JavaScript wrapper for browser. We attached payloads, such as that in Fig. 7, to the tangle using the restricted MAM mode. This allowed us to test how a patient could alter access controls to a particular data stream by updating their authorization key. After a transaction was confirmed on the tangle, we retrieved the data using the channel ID and authorization key.

To further explore MAM and assess its usability, we characterized performance of the current implementation of the MAM module. We broadcasted 300 payloads of size 1, 10, 100, 500 and 1000 characters in restricted mode using the MAM JavaScript wrapper for NodeJS on two different processors: Intel i7-7700 HQ @ 2.80 GHz, and ARMv7 Processor rev 5 (v7l). We chose these two processors because they serve as reference points for processors used in electronic and mobile healthcare. In particular, we chose the ARMv7 processor to model an Internet of Medical Things device.

## 4 Theory

### 4.1 Authenticated Data Structures

One-way hash functions are one of the fundamental building blocks of modern cryptography. They are used towards verification of data integrity, as well as message authentication codes and digital signatures. As such they represent the most important security primitive for the MAM extension module. Authenticated data structures have been well studied in the context of certificate revocation and validation, outsourced databases, sensor networks, and multicast packet authentication [27, [37], [38], [39]]. Source authentication ensures a receiver that the message originates from the claimed sender, and data authentication ensures that the data from that sender was unchanged. The notion of authentication in MAM includes both source and data authentication, ensuring both integrity and provenance in data exchanges.

The original approach to authenticated data structures builds upon Merkle Hash Trees, which were initially used for the purpose of one-time signatures and in Public Key Infrastructures to provide authentic and secure certificate revocation [40]. More advanced approaches to authenticated data structures apply hashing schemes for graph-structured data models, such as those used in biological and healthcare data [41]. In the context of this work we focus on MAM, which leverages Merkle Hash Trees to generate one-time signatures that authenticate data streams broadcasted from sensors. These data feeds do not lend themselves to be graph-structured, but may be part of an overarching graph.

### 4.2 Merkle Hash Technique

**Fig. 8:**
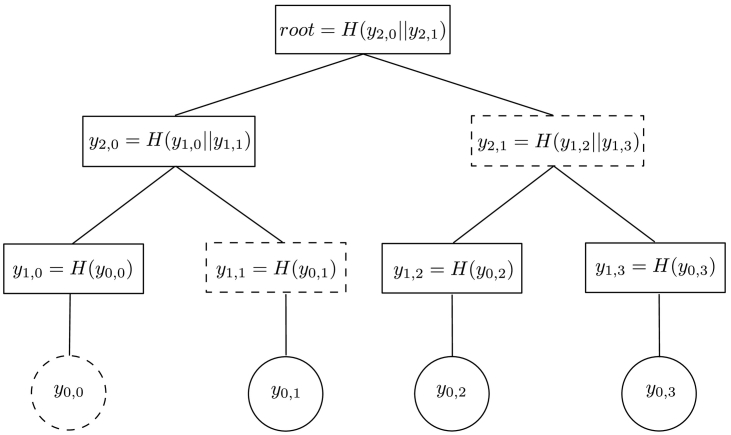
A binary Merkle Hash Tree constructed for authentic values *y*_0, 0_, *y*_0, 1_, *y*_0, 2_, *y*_0, 3_. The node values needed to verify *y*_0, 0_ are enclosed with dashed borders.

The Merkle Hash Technique generates hashing trees from the bottom-up [42]. A Merkle Hash Tree (MHT) intended for authentic values *y*_0, 0_, *y*_0, 1_, …, *y*_0, *n*_ is constructed by building a tree in which the leaves correspond to the hashes of the values of the ordered elements in the set. Therefore, a leaf associated with the element *y*_0, *i*_ contains the value *y*_1, *i*_ = *H*(*y*_0, *i*_), where *H*() is a cryptographic one-way hash function. The value of a node with multiple incoming edges corresponds to the hash of the concatenation of its predecessor nodes, maintaining their order. An internal node *y*_2, 0_ with children *y*_1, 0_ and *y*_1, 1_ therefore has the value *y*_2, 0_ = *H*(*y*_1, 0_‖*y*_1, 1_). An MHT can be used to prove the existence of an element in the set with the help of a verification object. A verification object contains a set of nodes which help the verifier in recomputing the root of the MHT. With this, the verifier can compute the root of the MHT and compare it to the publicly known root. For example, if we search for *y*_0, 0_ in the MHT in Fig. 8, the verification object contains node values *y*_0, 0_, *y*_1, 1_, and *y*_2, 1_. The verifier constructs *y*_1, 0_ = *H*(*y*_0, 0_), *y*_2, 0_ = *H*(*y*_1, 0_‖*y*_1, 1_) and finally *root* = *H*(*y*_2, 0_‖*y*_2, 1_). They can then verify the computed root by comparing it to the public value [38].

### 4.3 One-Time Signatures and the Merkle Signature Scheme

A one-time signature (OTS) scheme is a digital signature scheme that can only be used to sign one message per key pair. OTS schemes are based on one-way hash functions and enable faster signing and verification algorithms when compared to public key digital signature schemes such as RSA. However, OTS schemes are limited by the number of signatures that can be signed, the length of the signatures and size of the keys. The Merkle signature scheme (MSS), which is based on MHTs, provides a way to use one public verification key to verify multiple OTS.

Each leaf in the MHT corresponds to one OTS scheme. This means that each tree can produce the same number of messages as leafs in the MHT. These messages will all be verified using the public verification keys from the OTS scheme. The public verification keys of the OTS scheme are in turn verified by computing the root of the MHT from a given verification object, as shown in Fig. 8.

The MSS can be used to sign a limited number of messages with one public key, *pk*. The number of possible messages must be a power of two, so that we denote the possible number of messages as *N* = 2^*n*^. The first step of generating the public key *pk* is to generate the public keys *Y*_*i*_ and private keys *X*_*i*_ of 2^*n*^ one-time signatures. For each public key *Y*_*i*_, with 1 ≤ *i* ≤ 2^*n*^, a hash value *H*(*Y*_*i*_) is computed. With these hash values *h*_*i*_ an MHT is constructed. A tree with 2^*n*^ leafs and 2^*n*+1^ − 1 nodes is constructed. The root of the tree is the public key, *pk*, of the Merkle signature scheme.

To sign a message *M* with the MSS, the message *M* is signed with a one-time signature scheme, resulting in a signature *sig*^′^, first. This is executed by using one of the private and public key pairs (*X*_*i*_, *Y*_*i*_). Each leaf of the MHT is the hash of a one-time public key *Y*_*i*_. We denote *P* as the path from a given leaf to the root. The path *P* consists of *n* + 1 nodes, *P*_1_, …, *P*_*n*+1_, with *P*_1_ = *y*_1, *i*_ being the leaf and *P*_*n*_ = *y*_*n*+1, 0_ = *pk* being the root of the tree. To compute *P*, we need every child of the nodes *P*_2_, …, *P*_*n*+1_. We know that *P*_*i*_ is a child of *P*_*i*+1_. To calculate the next node *P*_*i*+1_ of the path *A*, we need to know both children of *P*_*i*+1_. We need the sibling node of *P*_*i*_ to complete this calculation. We refer to the sibling as *sib*_*i*_, so that *P*_*i*+1_ = *H*(*P*_*i*_‖*sib*_*i*_) in the case that *sib*_*i*_ is odd, *P*_*i*+1_ = *H*(*sib*_*i*_‖*P*_*i*_) if it is even. Hence, *n* nodes are needed to compute every node of the path *P*. The nodes, plus the one-time signature *sig*^′^ of *M* comprise the signature *sig* = (*sig*^′^‖*sib*_2_‖*sib*_3_‖*sib*_*n*−1_) of the MSS.

The receiver knows the public key *pk*, the message *M*, and the signature *sig* = (*sig*^′^‖*sib*_2_‖*sib*_3_‖*sib*_*n*−1_). At first, the receiver verifies the one-time signature *sig*^′^ of the message *M*. The receiver computes *P*_1_ = *H*(*Y*_*i*_) by hashing the public key of the one-time signature. For *l* = 1, …, *n* − 1, the nodes of *P*_*l*_ of the path *P* are computed with *P*_*l*_ = *H*(*y*_*l*−1_‖*sib*_*l*−1_) if the sibling index is odd, *P*_*l*_ = *H*(*sib*_*l*−1_‖*y*_*l*−1_) if even. If *P*_*n*_ = *pk* of the MSS, the signature is valid.

## 5 Results

In this case study we demonstrated that it was possible to use a distributed ledger to broadcast and receive authenticated, encrypted activity data from a wearable device. The source and integrity of the data were authenticated through the MAM module, and the data was structured using FHIR and coded with LOINC. This enabled structural and semantic interoperability across a diverse digital healthcare ecosystem with many potential stakeholders. In addition, we changed authentication keys during a broadcast stream to demonstrate how a patient could revoke access to future data they generate. This showed how granular, patient-defined access controls may work in the future.

**Fig. 9:**
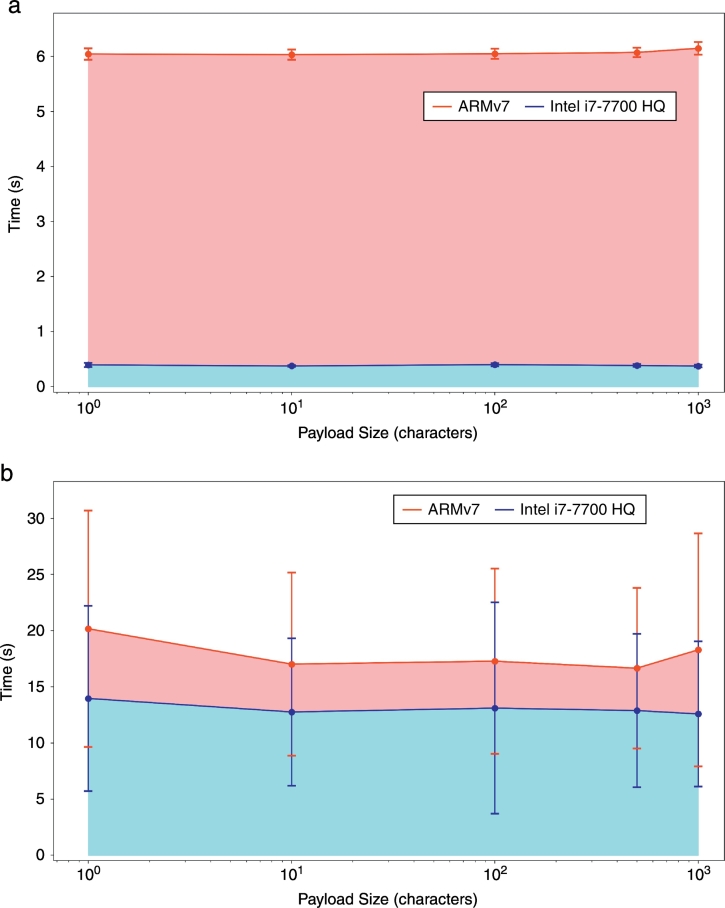
Latency for one **(a)** create or **(b)** attach cycle as a function of payload size and processor.

We characterized performance of the current implementation of MAM using an Intel i7–7700 HQ @ 2.80 GHz, and ARMv7 processor as stated in Section 3. Each point represents the average time of 300 trials for a given payload size, with error bars that signify the standard deviation from the mean. The results of these performance tests are given in Table 2 and displayed in Fig. 9.

**Table 2:** MAM experiment results. Time trials for create and attach actions using ARMv7 and Intel i7-7700 HQ processors. Payloads of size 1, 10, 100, 500, and 1000 characters were broadcasted to the tangle 300 times using each processor.

Processor	Action	Payload size (*chars*)	Trials	Avg. time (*s*)	St. dev. (*s*)	Variance (*s*^2^)	Min. (*s*)	Max. (*s*)
ARMv7 Processor rev 5 (v7l)	Create	1	300	6.0449	0.1035	0.0107	5.8730	6.7430
ARMv7 Processor rev 5 (v7l)	Create	10	300	6.0338	0.0927	0.0086	5.8800	6.7080
ARMv7 Processor rev 5 (v7l)	Create	100	300	6.0482	0.0931	0.0087	5.8260	6.8360
ARMv7 Processor rev 5 (v7l)	Create	500	300	6.0734	0.0861	0.0074	5.8540	6.9530
ARMv7 Processor rev 5 (v7l)	Create	1000	300	6.1480	0.1149	0.0132	5.9310	6.9960
Intel i7-7700 HQ @ 2.80 GHz	Create	1	300	0.3960	0.0370	0.0014	0.3540	0.5690
Intel i7-7700 HQ @ 2.80 GHz	Create	10	300	0.3775	0.0149	0.0002	0.3510	0.4550
Intel i7-7700 HQ @ 2.80 GHz	Create	100	300	0.4003	0.0300	0.0009	0.3600	0.6910
Intel i7–7700 HQ @ 2.80 GHz	Create	500	300	0.3861	0.0245	0.0006	0.3490	0.5190
Intel i7-7700 HQ @ 2.80 GHz	Create	1000	300	0.3761	0.0247	0.0006	0.3410	0.6400
ARMv7 Processor rev 5 (v7l)	Attach	1	300	20.1666	10.5214	110.7004	4.2210	69.6150
ARMv7 Processor rev 5 (v7l)	Attach	10	300	17.0180	8.1468	66.3711	2.6300	76.9440
ARMv7 Processor rev 5 (v7l)	Attach	100	300	17.2789	8.2522	68.0980	3.9790	73.9630
ARMv7 Processor rev 5 (v7l)	Attach	500	300	16.6553	7.1482	51.0970	3.7870	39.7320
ARMv7 Processor rev 5 (v7l)	Attach	1000	300	18.2881	10.3675	107.4854	3.5950	110.2530
Intel i7–7700 HQ @ 2.80 GHz	Attach	1	300	13.9647	8.2427	67.9413	1.016	45.2530
Intel i7-7700 HQ @ 2.80 GHz	Attach	10	300	12.7590	6.5611	43.0474	1.9910	32.4600
Intel i7-7700 HQ @ 2.80 GHz	Attach	100	300	13.1024	9.4090	88.5302	1.6540	125.2020
Intel i7-7700 HQ @ 2.80 GHz	Attach	500	300	12.8825	6.8178	46.4822	0.5340	35.6770
Intel i7-7700 HQ @ 2.80 GHz	Attach	1000	300	12.5842	6.4640	41.7834	0.0290	38.2030

The time required to change the MAM mode and convert the message from bytes to trytes was less than 1 ms. Therefore, we focused our analysis on the steps of broadcasting a message that had significant time delays. The two steps that we analyzed were *create* and *attach*. Both required non-negligible time to execute on the aforementioned processors.

We found that the time to *create* a message was well-defined, and dependent on the processor. However, the create time did not show any dependence on the payload size. The average time to *attach* a message displayed a high variance with no correlation to payload size. The i7–7700 processor was correlated with a lower average *attach* time than the ARMv7 processor across our trials. However, the wide range of times for both processors prevent us from claiming the average *attach* time differs with statistical significance. This behavior is to be expected since the attach step includes a small amount of proof-of-work, which is inherently a random process. Neither the i7–7700 nor ARMv7 processors were optimized to perform the proof-of-work algorithm in the IOTA protocol, so our observations of highly dispersed *attach* time intervals are in agreement with the expected behavior.

## 6 Discussion

Activity data generated outside the supervision of healthcare professionals can provide a more complete narrative to assist in patient care from remote locations. Establishing an encrypted, distributed data store with access controls governed by the citizen has the potential to create a more comprehensive record. This would enable predictive models based on population level health data, with proper consent.

IOTA is a permissionless distributed ledger protocol that provides the *fabric* for an immutable audit trail of activity data broadcasted from wearable devices. MAM is an extension module housed in the *application* layer of the IOTA stack, and has the potential to enable patients to store, retrieve, and share their authenticated, encrypted activity data on-demand via the tangle. This communication protocol empowers patients by giving them agency over their activity data, and allows them to be better informed of their current status and prior trends. The restricted mode of MAM gives patients granular controls over the way their data is exchanged between participants in the digital healthcare ecosystem, while the added layer of integrity from the tangle establishes trust that the data has not been altered. In the remainder of this section we discuss the security, privacy, interoperability, and feasibility of our implementation.

Our implementation leverages interoperability standards that are based on modern web services, and inherits the key elements of distributed ledger technologies. This model leads to improved security with the encryption and access control features from MAM. MAM relies on the distributed nodes participating in the IOTA network to avoid a single point of failure. All encrypted data is added to the ledger stored on each full node. Since the encrypted activity data remains distributed, our system does not create a central target for a cyberattack or data leak. It is important to state that our implementation gives the patient agency over their authorization keys. This enables patients to define the level of control they want over their data - for instance, some patients may not want to be responsible for maintaining their medical data, while others may want to be in full control. This also brings about its own challenges as these keys must be managed properly by the patient, their guardian, a designated care provider, or a third party.

Use of distributed ledger technologies seems to counter our current notion of digital privacy since all nodes in a network must contain a copy of the ledger's current state. Although value-based transactions on distributed ledgers are pseudonymous, a thorough analysis of transaction frequencies and points of origin could allow one to infer that an entity has repeatedly interacted with another party by analyzing network traffic. Furthermore, it is even possible to infer how many tokens an entity holds with varying levels of uncertainty [43]. Improving privacy while preserving auditability on a distributed ledger is an ongoing area of exploration. However, this issue is not a problem for our implementation because MAM removes the notion of two entities interacting with each other. Rather, the transaction addresses contained in a data stream are randomly generated by the issuer, and independent of the parties that have the details needed to fetch this encrypted data. It is important to note that subscribers to a public or private stream will be able to follow the stream from their point of entry onwards due to the next channel key being embedded in the current message. However, our implementation uses the restricted mode of MAM which grants a patient the ability to change their authorization key and make the addresses of future transactions in a stream unpredictable for past subscribers as described in Section 2.3. In contrast to the transparency of traditional distributed ledgers, the MAM channel modes provide access controls that can make data contained within transactions private. This is possible because of authorization keys, which provide granular access controls to segments of an activity data stream. Authorization keys could be generated and stored by a patient if they choose, or left to the discretion of healthcare professionals if patients do not want that level of control over their own data. To plan for emergencies, it is logical for these authorization keys to be stored with a Regional Health Information Organization (RHIO), or another governmental body. This will ensure that healthcare providers could access data in cases when the patient is unconscious, or otherwise unable to recall or provide a log of their authorization keys.

To address interoperability across the digital healthcare ecosystem, we chose FHIR and LOINC as the structural and semantic layers of interoperability for our activity data implementation. Structuring activity data in this format will enable participants across the digital healthcare ecosystem to interpret or map the data and extract actionable meaning. With this level of semantic interoperability, activity data can be seamlessly shared between parties with proper permissions. This can open the door to harnessing the power of big data analytics to improve population level models, that predict the likelihood of future health events for individual patients, based on their current status.

Although we demonstrated how MAM could be used with FHIR and LOINC, we designed our framework with the flexibility to support all open standards for health data exchange. Furthermore, MAM is able to transmit data from any endpoint that has a link to the internet, whether that be a physician's computer, a hospital server, a mobile device, or a bluetooth low energy sensor embedded in a patient. Since encrypted data stored on the tangle is accessible through open APIs, our implementation can be seamlessly integrated with any stakeholder in the digital healthcare ecosystem. We believe this will ease adoption and open the door for use cases beyond activity data collected outside of the supervision of healthcare professionals.

To test the feasibility of MAM as a conduit for interoperable activity data, we tested current performance of the MAM library. We found that this library is not optimized, and needs to be improved for use in healthcare workflows. There are multiple components associated with these performance issues. With regards to CPU, there are currently improvements being implemented to significantly reduce the times for the *create* and *attach* steps. We should note that the *attach* step requires a small amount of proof-of-work, and therefore has a baseline level of randomness associated with it. While this randomness is healthy for the network as a whole, the implementation can be optimized to run more efficiently on different CPUs. Also important are network latency issues, but these are out of the scope of this report.

## 7 Future Work

As we look to take our implementation of MAM from a research prototype to a protocol for healthcare data exchange between all stakeholders in the digital healthcare ecosystem, we have identified several key areas for future work. To improve the MAM-based system, we will engage with healthcare stakeholders across the ecosystem, from patients and providers, to hospital IT departments, pharmaceutical companies, insurance companies, government institutions, and more. We are currently in the process of addressing functionality requirements and additional use case scenarios to provide feedback for future improvements of the MAM protocol. For instance, we have suggested that a secure key exchange protocol should be integrated within the MAM module to swap authorization keys between parties.

As the MAM module matures, we will develop a proof-of-concept across academic institutions to demonstrate how this technology could be used to authenticate data streams when remotely monitoring patients, with minimal disruption of their day-to-day activities. While both the *fabric* layer of IOTA and modules such as MAM that sit in the *application* layer will be open source and governed in a distributed manner, further customizations of MAM can be proprietary and optimized for a particular use case. This modularity will provide us with the capability to continuously use and improve MAM for our use-case irrespective of the *fabric* layer.

An area of interest within this proof-of-concept is addressing emergency situations where a patient arrives in an emergency room unconscious or in distress. It is imperative that healthcare professionals can access the patient's activity data without requiring the patient to recall or provide an authorization key. This is a difficult problem to address, but we have plans to explore secure biometric identity solutions such as palm vein, iris, and voice scanners. Establishing an on-demand identity solution across healthcare specific nodes that are distributed across providers, organizations and RHIOs may alleviate this issue. To find such an on-demand identity solution that is secure, we will explore contemporary web standards for authorization such as OAuth, and the related works that have attempted to integrate these standards into the healthcare IT framework [44].

Lastly, since such a distributed data store could grow exponentially as more patients adopt this technology as a solution, we need to address how such a large data set could be maintained across all stakeholders. While the tangle currently scales due to its modest activity, the world of embedded and wearable devices has the potential to produce volumes of data that will not suit the current notion that every full node in the network stores the entire history. In the future, full nodes will store the current state, and will prune the remainder of the data to restore capacity to handle new transactions. These pruned transactions will still have verifiable cryptographic relationships, but the complete history of relevant transactions will need to be maintained. For instance, healthcare nodes will need to know which transactions to store locally, and which to discard in the pruning process. This process will happen within local networks, so that every provider need not maintain a copy of every healthcare transaction, just the ones relevant to their registered patients. However, a RHIO, or other organization, may be responsible for maintaining a complete history of the patients within their jurisdiction. This can serve as a source of truth to validate that data being accessed across institutions is authentic.

## 8 Conclusions

This report explored the emergence of an on-demand digital healthcare ecosystem that algorithmically processes large volumes of data, and addressed the need for this data to be authenticated, distributed, and immutable. We demonstrated how distributed ledger technologies can play a key role in ensuring authenticity of encrypted activity data by using the MAM module of the IOTA protocol. This module also enabled patients to define granular access controls that could be updated over time. While the current implementation of MAM proved to be an effective conduit for authenticated activity data, it has room for design and performance improvements. Such a method for secure and effective coordination of interoperable activity data can open the door for remote monitoring, and other on-demand services that catapult healthcare into the digital age.

## Footnotes

1 JavaScript Object Notation (JSON), REpresentational State Transfer (REST), HyperText Transfer Protocol (HTTP).

2 The current implementation of IOTA uses balanced ternary.

3 We broadcasted heart rate data from an Apple Watch.
